# Melatonin Enhances Blast Disease Resistance via Inducing Rice Immunity and Inhibits the Growth of the *Magnaporthe Oryzae*

**DOI:** 10.1186/s12284-025-00824-1

**Published:** 2025-07-19

**Authors:** Si-Jia Yang, Xiu-Lian Yan, Mao-Lin Guo, Ya-Ping Tang, Rong Liao, Xiao-Xiao Yin, Beenish Hassan, Ming Yuan, Zhi-Xue Zhao, Wen-Ming Wang

**Affiliations:** 1 State Key Laboratory of Crop Gene Exploration and Utilization in Southwest China, Sichuan Agricultural University, Chengdu, 611130 China; 2 College of Life Science, Sichuan Agricultural University, Ya’an, 625014 China

**Keywords:** Rice, Melatonin, *Magnaporthe oryzae*, Immunity, Biopesticides

## Abstract

Biopesticides are promising alternatives to chemical pesticides because of their low residual effects, high selectivity, and capacity for long-term disease control. Melatonin (*N*-acetyl-5-methoxytryptamine) may be an ideal candidate for biopesticide because it is widely present in the plant kingdom, involved in growth, development, and stress-induced responses in plants, and can inhibit the growth and propagation of some microbial pathogens. However, it remains largely unclear whether melatonin influences rice and the blast fungus *Magnaporthe oryzae*. Here, we demonstrate that melatonin enhances rice immunity and inhibits the growth of *M. oryzae*, resulting in resistance to rice blast disease. Melatonin acts in rice response to *M. oryzae* because biosynthesis-related genes are induced upon *M. oryzae* infection. Melatonin treatment remarkably reduces blast disease severity in a susceptible rice accession. Mechanistically, melatonin treatment activates the mitogen-activated protein kinase cascades and up-regulates the expression of defense-related genes. Melatonin treatment also significantly inhibits the growth, sporulation, and spore germination of *M. oryzae*. Notably, melatonin treatment results in the death of *M. oryza*e hyphal cells. Altogether, our findings indicate that melatonin plays dual roles in the rice-*M. oryzae* interactions, activating rice immunity and inhibiting fungal growth. Thus, this study offers insights into the potential development of novel melatonin-based biopesticides for controlling rice blast disease.

**Supplementary Information:**

The online version contains supplementary material available at 10.1186/s12284-025-00824-1.

## Background

Rice blast disease is caused by the fungal pathogen *M. oryzae* and severely threatens rice production worldwide. Reports indicate that rice blast disease usually results in a 10–30% yield loss (Zhang et al. 2022), and in severe cases, may lead to complete crop failure, threatening global food security (Mutiga et al. 2021; Islam et al. 2023). *M. oryzae* conidia germinate on the leaf surface to form a specialized osmotic structure called an appressorium. By accumulating osmotic substances, the appressorium produces turgor pressure and then penetrates the rice cuticle and cell wall, and forms invasive mycelia to pillage nutrients from the host cells while concomitantly secreting effectors that interfere with the host’s immunity (Huang et al. 2020; Zhang et al. 2024). Initially, *M. oryzae* exhibits biotrophic growth, feeding on living tissues before transitioning to necrotrophic growth, which involves the secretion of toxins and degradative enzymes to kill host cells (Cruz-Mireles et al. 2021; Islam et al. 2023). Timely control of this disease upon its epidemics mainly relies on the application of chemical pesticides that are widely employed in rice-producing regions (Akter et al. 2023). For instance, mycelium growth and spore germination of *M. oryzae* can be significantly inhibited by applying 0.006–0.056 µg mL^−1^ azoxystrobin or 0.024–0.287 µg mL^−1^ kresoxim-methyl (Chen et al. 2015). Combining azoxystrobin with silver nanoparticles (AgNPs) enhances the inhibitory effects on both azoxystrobin-sensitive and resistant *M. oryzae* strains (Shi et al. 2023). Despite their effectiveness in controlling *M. oryzae* infection, chemical pesticides raise environmental concerns and pesticide resistance (Gulshan and Moye-Rowley 2007). Thus, researchers have been testing various biopesticides that can mitigate the issues associated with chemical pesticides, leading to the identification and utilization of chitin and its derivatives, benzophenanthridine alkaloid sanguinarine (SAN), and *Lysimachia foenum-graecum* Herba extract (Lee 2016; Hezakiel et al. 2023; Zhao et al. 2023). To date, biopesticides have gained increasing attention due to their desirable attributes, including low residual effects, high selectivity, and capacity for long-term disease control (Meshram et al. 2022; Lei et al. 2023). For example, chitin and its derivatives enhance plant resistance to pathogens by influencing plant metabolic pathways (Velásquez and Pirela 2016; Giraldo et al. 2023). SAN significantly inhibits the *M. oryzae* mycelial trophic growth and appressorium formation (Anjago et al. 2021). The *Lysimachia foenum-graecum* Herba extract inhibits the growth of *M. oryzae* mycelia (Lee 2016). Biopesticides used for pathogen control generally fall into two main categories. The first category enhances plant immunity against pathogens. For example, chitin-derived biopesticides trigger plant immune responses and are most effective when applied before pathogen invasion (Kramer and Muthurkishnan 1997). The second category inhibits pathogen growth. For example, validamycin disrupts pathogen development and is most effective when applied at the initial stage of infection (Peng et al. 2014). However, biopesticides that simultaneously enhance plant immunity and inhibit pathogen growth are extremely rare.

Melatonin is a pleiotropic molecule with various functions in plants and microbes, primarily functioning as an antioxidant that mitigates the effects of reactive oxygen species (ROS) and other harmful oxidative molecules (García et al. 2014; Gibas et al. 2014; Lee et al. 2015; Arnao and Hernández-Ruiz 2017). Additionally, melatonin is involved in plant growth and development and responses to both abiotic and biotic stresses. First, melatonin participates in multiple processes of plant growth and development, including flower development (Lee et al. 2019), root growth (Yang et al. 2021), fruit ripening (Wang et al. 2020), and leaf senescence (Wang et al. 2013). Second, melatonin is involved in abiotic stress. It promotes plant growth under cold stress by upregulating the expression of C-repeat binding factors (CBFs)/Drought Response Element Binding factors (DREBs), *cold-regulated 15a* (*COR15a*), *calmodulin-binding transcription activator 1* (*CAMTA1*), *C*_*2*_*H*_*2*_*-type zinc finger transcription factor 10* (*ZAT10*), and *ZAT12* (Bajwa et al. 2014). Under high salinity, melatonin reduces oxidative damage by scavenging H_2_O_2_ or enhancing the activities of antioxidant enzymes, such as ascorbate peroxidase, catalase, and peroxidase. Moreover, melatonin significantly upregulates the expression of ion channel genes *MdNHX1* and *MdAKT1* of *Malus hupehensis* Rehd, contributing to the maintenance of ion homeostasis and enhancing plant salt tolerance (Li et al. 2012). Finally, melatonin plays a crucial role in biotic stress responses, interacting with plant innate immune signaling and hormone signaling pathways, such as salicylic acid (SA), jasmonic acid (JA), and ethylene (ET), to enhance plant defense against pathogenic microbes (Sun et al. 2021; Mansoor et al. 2024). Specifically, melatonin activates MKK4/5/7/9, leading to the activation of MPK3 and MPK6, which in turn activate a series of transcription factors, thereby inducing the expression of various defense-related genes (Lee and Back 2016). Exogenous application of melatonin enhances rice immunity against *Xanthomonas oryzae* pv. *oryzae* and *M. oryzae* by increasing nitric oxide synthase, nitrate reductase, and/or peroxidase activity (Chen et al. 2020; Yuan et al. 2025). Moreover, the melatonin synthesis pathway mutant *snat*, which has significantly reduced melatonin levels, exhibits decreased SA levels and increased sensitivity to pathogen infection. Exogenous melatonin treatment restores the expression of defense-related genes in the *snat* knockout mutant but not in *nahG* Arabidopsis plants, suggesting that melatonin-induced defense responses are SA-dependent (Lee et al. 2015). Furthermore, defense-related gene expression induced by melatonin is partially or completely suppressed in *npr1*, *ein2*, and *mapk6* mutants, indicating that melatonin-mediated defense signaling requires not only SA but also ET (Lee et al. 2014). These findings collectively highlight melatonin’s role as a novel signaling molecule in plant-pathogen interactions. Besides, melatonin exhibits fungicidal activity by inhibiting the growth of various plant pathogens. Different concentrations of melatonin have been shown to suppress the growth of fungal pathogens such as *Alternaria* spp., *Botrytis* spp., and *Fusarium* spp. (Arnao and Hernández-Ruiz 2015). Exogenous melatonin application has also been reported to reduce the lipid droplets in *Phytophthora infestans*, altering its mycelial cell ultrastructure and inhibiting its growth (Zhang et al. 2017). Moreover, a few studies have demonstrated the inhibitory effects of melatonin on pathogenic fungi, bacteria, and viruses (Arnao and Hernández-Ruiz 2015; Lee and Back 2017; Lu et al. 2019; Li et al. 2022). However, the role of melatonin in the interaction between rice and the rice blast fungus remains largely unclear.

In this study, to test whether melatonin has any effects on rice immunity against *M*. *oryzae*, we treated rice susceptible accession Lijiangxin Tuan Heigu (LTH) with different concentrations of melatonin and performed blast disease assays. We then conducted western blotting and RT-qPCR to assess the activation of MAPK cascade and the expression of defense-related genes. We also tested whether melatonin has any effects on the mycelial growth, conidial germination, and mycelial death of *M. oryzae* with varying melatonin concentrations applied to *M. oryzae* strain GZ8. Our data showed that melatonin holds great potential as a natural biocontrol agent against *M. oryzae*.

## Materials and Methods

### Plant Material, Rice Blast Strain, and Cultural Conditions

Lijiangxin Tuan Heigu (LTH), Q455, and the *oscebip oscerk1* double mutant were used in this study; Q455 and the *oscebip oscerk1* double mutant were obtained from a previous study (Li et al. 2024). Seedlings were cultivated in a growth chamber at 26 ºC and 70% relative humidity, with a photoperiod of 12 h of light and 12 h of darkness. *M. oryzae* isolates, including Zhong10-8–14 and eGFP-tagged Zhong10-8-14 (GZ8) (generously provided by Prof. Lihuang Zhu, Chinese Academy of Sciences), were cultured on complete medium (CM) in a light incubator with a photoperiod of 16 h of light and 8 h of darkness at 28 ºC for approximately 10 days. The hyphae were then scratched and further cultivated at 28 ºC under continuous light for 4 days to promote sporulation. The Zhong10-8-14 strain was used for reactive oxygen species (ROS) detection, whereas GZ8 was used for all other experiments.

### Rice Blast Inoculation

The rice blast inoculation was conducted as previously described (Zhao et al. 2020). Briefly, the detached leaf sections of three-leaf-stage seedlings were placed on the surface of the aqueous solution containing 0, 0.025, 0.1, and 0.5 mM melatonin, respectively, and were subsequently drop-inoculated with 5 μL of spore suspension (1 × 10^5^ spores/mL) at wounded sites on each leaf. Disease symptoms were observed at 5 days post-inoculation (dpi). Image J was used to calculate the lesion area. GraphPad Prism was employed to analyze the correlation between lesion area and melatonin concentration.

### Expression Analysis of Melatonin Biosynthesis-Related Genes in Rice During *M. Oryzae* Infection

The RNA-seq data of five rice accessions before and after *M. oryzae* inoculation were obtained from a previous study (Hu et al. 2023). To determine whether *M. oryzae* infection affects the expression of genes involved in melatonin biosynthesis, we analyzed transcriptome data from samples collected at 0 and 24 h post-inoculation (hpi) because *M. oryzae* forms the appressorium to invade through the rice epidermis at 24 hpi (Martin-Urdiroz et al. 2016; Du et al. 2021). Gene expression levels at 24 hpi were normalized to those at 0 hpi, and a heatmap was constructed to visualize expression changes. To confirm whether the expression of melatonin biosynthesis-related genes observed in the transcriptome data accurately reflects induction by *M. oryzae*, LTH at the three-leaf stage were inoculated with *M. oryzae* at the indicated time points. Leaf samples were collected and subsequently processed for total RNA extraction and cDNA synthesis as previously described (Zhao et al. 2022). A real-time quantitative polymerase chain reaction (RT-qPCR) was then performed to analyze the expression of melatonin synthesis-related genes (Bhowal et al. 2021), including *OsTDC4*, *OsTDC5*, *OsT5H1*, *OsSNAT1*, *OsASMT13*, and *OsASMT15*. The abundance of detected genes was normalized using the *ubiquitin* (*UBI*) gene as an internal standard. All primers used for RT-qPCR are listed in supplementary table S1.

### Detection of Defense-Related Gene Expression

The detached leaf sections of three-leaf-stage LTH seedlings were placed on the surface of the aqueous solution containing 0, 0.025, 0.1, and 0.5 mM melatonin. The leaves treated with different concentrations of melatonin were collected at 0, 6, and 12 h, and were subsequently subjected to total RNA extraction and cDNA preparation as previously described (Zhao et al. 2022). RT-qPCR was then performed to analyze the expression patterns of several defense-related genes, including *chitin elicitor-binding protein* (*OsCEBiP*, Os03g0133400), *pathogenesis-related protein 1a* (*OsPR1a*, Os07g0129200), *pathogenesis-related gene 10* (*OsPR10*, Os12g0555000), *probenazole inducible gene 1* (*OsPBZ1*, Os12g0555500), *NAC domain-containing protein 4* (*OsNAC4*, Os01g0816100), and *jasmonate ZIM-domain protein 8* (*OsJAZ8*, Os09g0439200). The chitin-binding protein OsCEBiP, together with chitin elicitor receptor kinase 1 (OsCERK1), constitutes a functional receptor complex required for fungal chitin perception and mediates subsequent immunity in rice (Akamatsu et al. 2013). *OsPBZ1* functions as a defense marker gene in rice (Kim et al. 2008), while *OsNAC4* is a marker gene to measure the amplitudes of PTI (Hassan et al. 2025). *OsPR1a* and *OsPR10* are well-characterized defense-related genes associated with pathogen resistance (Hashimoto et al. 2004; Mitsuhara et al. 2008). *OsJAZ8* acts as a JA signal repressor in rice (Yamada et al. 2012). To determine whether melatonin-induced expression of defense-related genes depends on *OsCERK1* and *OsCEBiP*, we treated the leaves of a three-week-old *oscebip oscerk1* double mutant with H₂O, melatonin (0.5 mM), chitin (100 μg mL^−1^), and a combination of chitin (100 μg mL^−1^) and melatonin (0.5 mM). Wild-type rice accession Q455 served as the control. Leaf samples were harvested at 0 and 6 h post-treatment, followed by total RNA extraction and cDNA synthesis. RT-qPCR was performed to quantify the expression levels of *OsPBZ1*, *OsPR10*, and *OsNAC4*. The abundance of detected genes was normalized using the *ubiquitin* (*UBI*) gene as an internal standard. The relative expression level of genes was calculated using the 2^−△△Ct^ method (Livak and Schmittgen 2001). All primers used for RT-qPCR are listed in Supplementary Table S1.

### Kinase Assays Using the Plantlets

For the analysis of the melatonin-activated MAPK, rice seedlings were cultivated on a 1/2 MS medium containing 1% sucrose and then maintained at 26 °C with a photoperiod of 12 h of light followed by 12 h of darkness for approximately 10 days. Subsequently, the leaves of the seedlings were excised into 1 cm segments and incubated in Petri dishes containing distilled water for 12 h. The leaf segments were then treated with solutions containing 0 and 0.025 mM melatonin for 0, 15, and 30 min, respectively, and the samples were collected accordingly. Equal amounts of samples collected at different time points were processed for total protein extraction following the method previously reported (Zhao et al. 2021). Total proteins were separated by 10% SDS-PAGE and MAPK activation was detected using the Phospho-p44/42 MAPK antibody (CST). Quantitative analysis of the phosphorylated MAPK bands before and after melatonin treatment was performed using ImageJ software, and the data were graphically represented using GraphPad Prism.

### Phenotypic Characterization

To analyze the inhibitory effects of melatonin on the growth of *M. oryzae*, we inoculated 6 × 6 mm mycelial plugs onto a complete medium (CM), rice powder agar (RPA) medium, and potato dextrose agar (PDA) medium, each containing 0, 1, 2, and 4 mM melatonin, respectively. The cultures were incubated at 28 °C under a photoperiod of 16 h of light followed by 8 h of darkness for 8 days, after which the colony radii were measured. For analysis of the inhibitory effects of melatonin on the conidiation of *M. oryzae*, equal quantities of hyphae were inoculated onto CM, RPA, and PDA media containing either 0 mM or 4 mM melatonin and incubated at 28 °C under continuous light for 4 days. Spore production was observed using a fluorescence microscope (Axio Imager A2). Subsequently, the conidia were washed from the culture plates with equal volumes of sterile water, and their counts were determined using a hemocytometer. To analyze the inhibitory effects of melatonin on conidial germination, we inoculated the conidial suspensions of *M. oryzae* onto hydrophobic coverslips containing 0, 1, 2, and 4 mM melatonin, and incubated them at room temperature in darkness for 24 h. Conidial germination and appressorium formation were then observed and quantified.

### Dead Fungal Cell Staining and ROS Detection

Spores of the GZ8 or Zhong10-8–14 strain were inoculated into yeast extract peptone dextrose (YEPD) liquid medium and cultured at 28 °C for 4 days in a rotary shaker. Mycelia were then harvested and treated with either 0 or 4 mM melatonin for 8 h. Subsequently, the treated mycelia were collected and resuspended in PBS buffer. For the assessment of fungal cell death staining and reactive oxygen species (ROS) levels following melatonin treatment, hyphae of GZ8 and Zhong10-8–14 were incubated with 0.5 μM propidium iodide (PI) and 10 μM 2',7'-dichlorofluorescein diacetate (DCFH-DA) (Kong et al. 2021), respectively, for 30 min. The stained hyphae were washed 5–6 times with PBS and observed under a fluorescence microscope (Axio Imager A2). Dead fungal cells exhibited red fluorescence, while green fluorescence intensity increased proportionally with ROS accumulation.

### Statistical Analysis

Statistical analyses were conducted using IBM SPSS Statistics 20.0 (IBM Corp., Armonk, NY, USA). Pearson correlation analysis was employed to evaluate relationships between variables, with *R* representing the correlation coefficient. The effects of melatonin on *M. oryzae* conidiation were assessed by Student’s *t*-test, with significance indicated by *** for *P* < 0.001. All other datasets were analyzed by one-way ANOVA followed by Tukey’s multiple comparison test, where different letters indicate statistically significant differences (*P* < 0.05). All experiments were independently repeated at least three times, and results are presented as means ± standard deviation (SD).

## Results

### *M. Oryzae* Induces the Expression of Melatonin Biosynthesis-Related Genes

**Fig. 1:**
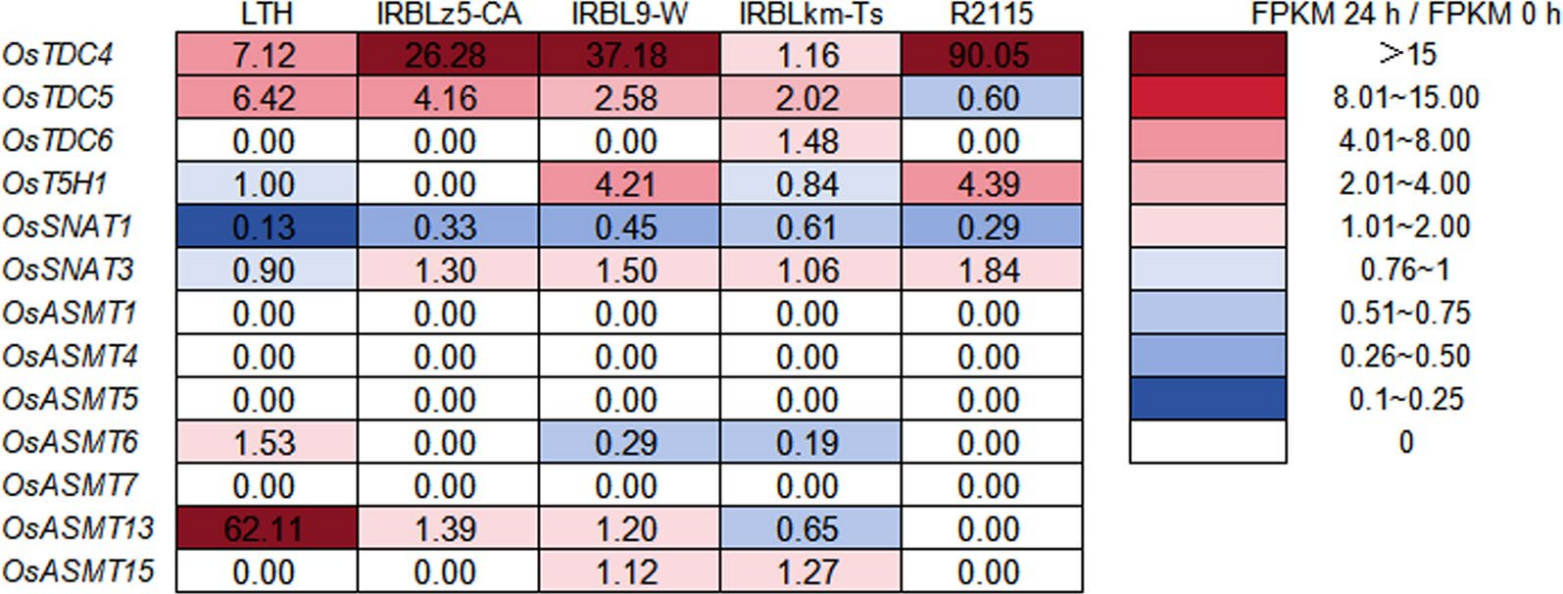
Heatmap depicting the expression of melatonin biosynthesis-related genes following *M. oryzae* inoculation in LTH, IRBLz5-CA, IRBL9-W, IRBLkm-Ts, and R2115. The number in each box represents the ratio of gene expression levels at 24 hpi to those before inoculation. IRBLz5-CA, IRBL9-W, and IRBLkm-Ts contain *Pi2*, *Pi9*, and *Pikm*, respectively. R2115 contains *Pi2*, *Pid2*, and *Pib*

To determine whether *M. oryzae* infection influences the expression of melatonin biosynthesis-related genes in rice, we examined RNA-seq data of rice accessions infected with *M. oryzae*, including the blast-susceptible accession LTH, monogenic lines carrying single resistance (*R*) genes, and the highly blast-resistant restorer line R2115. Upon *M. oryzae* infection, *OsTDC4* was induced across all five accessions (Fig. 1). *OsTDC5* was upregulated in LTH, IRBLz5-CA, IRBL9-W, and IRBLkm-Ts, whereas *OsTDC6* showed specific induction in IRBLkm-Ts. Additionally, *OsT5H1* was upregulated in IRBL9-W and R2115, whereas *OsSNAT3*, but not *OsSNAT1*, was upregulated in all accessions except LTH. Notably, *OsASMT6* was induced in LTH, *OsASMT13* was upregulated in LTH, IRBLz5-CA, and IRBL9-W, and *OsASMT15* showed increased expression in IRBL9-W and IRBLkm-Ts. To further validate the induction of melatonin biosynthesis-related genes by *M. oryzae* infection, we examined the expression of *OsTDC4*, *OsTDC5*, *OsT5H1*, *OsSNAT1*, *OsASMT13*, and *OsASMT15* following pathogen inoculation. As shown in Figure S1, *OsTDC4* expression was significantly upregulated at 72 and 120 hpi (Fig. S1a), while *OsTDC5* showed significant induction at 24, 72, and 144 hpi (Fig. S1b). *OsT5H1* expression was significantly induced at 72 hpi (Fig. S1c). *OsSNAT1* expression was also significantly induced at 48, 120, 144, and 168 hpi (Fig. S1d). *OsASMT13* exhibited significant upregulation at all time points except at 24 and 96 hpi (Fig. S1e). The expression of *OsASMT15* was significantly induced at all time points except for a slight increase at 120 hpi (Fig. S1f). Although some discrepancies were observed for certain genes at 24 hpi between RNA-Seq and RT-qPCR expression patterns, these differences may reflect the delayed transcriptional responses captured in the cDNA samples or from the use of independent experimental replicates. Nevertheless, *M. oryzae* infection differentially induces the upregulation of melatonin biosynthesis-related genes in different rice accessions, implying the involvement of melatonin in the interactions of rice with *M. oryzae*.

### Melatonin Treatment Reduces Rice Blast Disease Severity

**Fig. 2:**
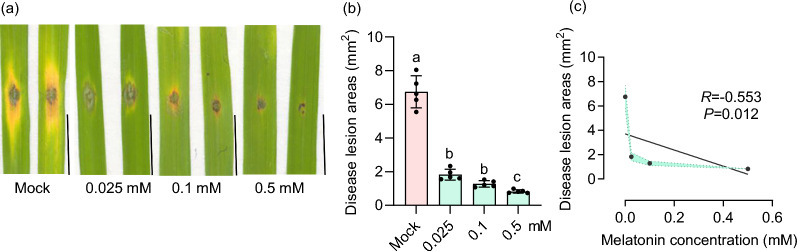
Melatonin treatment reduces rice blast disease severity. **a** Detached leaf sections of three-week-old rice seedlings were treated with different concentrations of melatonin and then inoculated with the GZ8 strain using punch inoculation. Photographs were taken at 5 days post-inoculation (dpi). **b** The quantification of disease lesion areas in **a**. The data are presented as means ± SE (n = 3). **c** Negative correlation between melatonin concentration and disease lesion area. The associated Pearson correlation coefficients are shown as *R*. The data are presented as means ± SE (n = 3). In this figure, lowercase letters indicate statistically significant differences (*P* < 0.05, one-way ANOVA with Tukey’s test). All experiments were performed three times with similar results

To investigate the role of melatonin in the interactions of rice with the blast fungus, we immersed the detached leaf sections of LTH in aqueous solutions containing 0, 0.025, 0.1, and 0.5 mM melatonin, followed by inoculation with *M. oryzae* strain GZ8. The blast disease phenotypes were then evaluated at five dpi. The results showed that treatment with 0.025 mM melatonin significantly reduced the size of disease lesions compared to the mock treatment. Notably, as the concentration of melatonin increased, the lesion size progressively decreased (Fig. 2a, b), indicating that melatonin reduces blast disease severity in a concentration-dependent manner. Consistently, the concentration of melatonin in the treatment was negatively correlated with the disease lesion areas (Fig. 2c). These results indicate that melatonin treatment remarkably reduces rice blast disease severity.

### Melatonin Treatment Enhances Rice Immune Responses

**Fig. 3:**
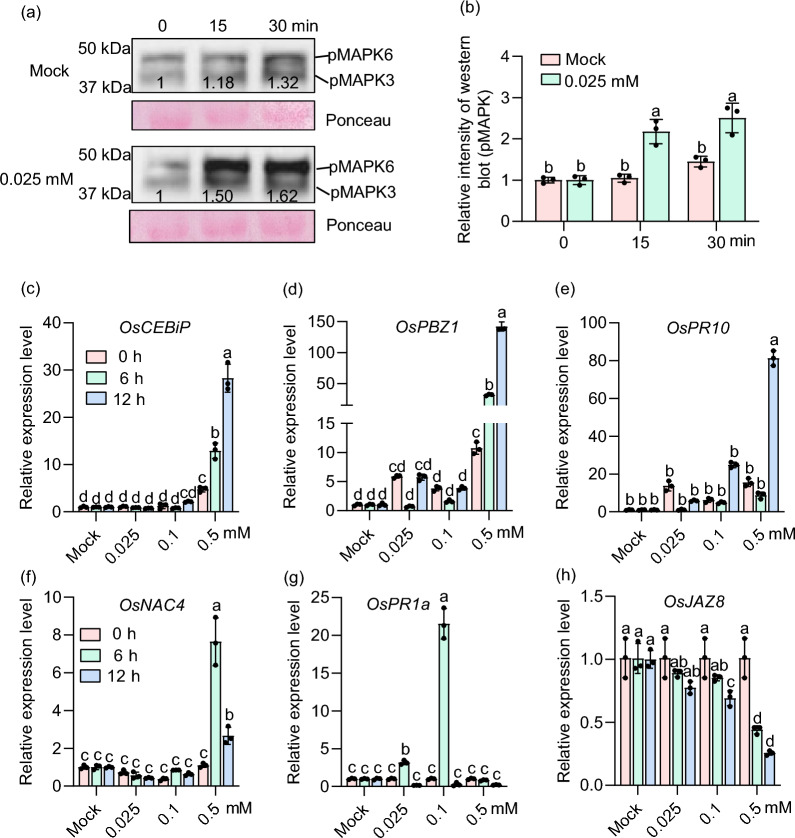
Melatonin induces MAPK activation and defense-related gene expressions in rice. **a** Melatonin-induced MAPK activation was analyzed by immunoblots with α-pMAPK. **b** The optical density of the phosphorylated MAPK bands was normalized to the corresponding loading control bands stained by Ponceau in **a**. The data are represented by means ± SE (n = 3). **c**–**h** RT-qPCR was employed to analyze the expression patterns of defense-related genes at 0, 6, and 12 h following treatment with 0, 0.025, 0.1, and 0.5 mM melatonin, including *OsCEBiP* (**c**), *OsPBZ1* (**d**), *OsPR10* (**e**), *OsNAC4* (**f**), *OsPR1a* (**g**), and *OsJAZ8* (**h**). The data are represented by means ± SE (n = 3). In this figure, lowercase letters indicate the statistically significant difference (*P* < 0.05, one-way ANOVA with Tukey’s test). All the experiments were conducted three times with similar results

To explore why melatonin treatment reduces rice blast disease severity, we examined immune responses upon melatonin treatment. Previous studies have demonstrated a link between melatonin and the activation of plant immune responses (Mansoor et al. 2024). In particular, melatonin has been shown to exert its biological effects through the mitogen-activated protein kinase (MAPK) cascade in Arabidopsis (Back 2021). Thus, we initially assessed the phosphorylation of MAPK3 and MAPK6 and observed that 0.025 mM melatonin treatment obviously increased the phosphorylation of these two MAPKs at 15 and 30 min (Fig. 3a, b). Then, we analyzed the expression patterns of defense-related marker genes, including *OsCEBiP*, *OsPBZ1*, *OsPR10*, *OsNAC4*, and *OsPR1a*, and a repressor of jasmonic acid (JA) signaling, *OsJAZ8* (Yamada et al. 2012; Yang et al. 2021). Compared to pre-treatment, the expression of *OsCEBiP*, *OsPBZ1*, and *OsNAC4* was significantly elevated at both 6 and 12 h after 0.5 mM melatonin treatment (Fig. 3c, d, and f). The expression of *OsPR10* was significantly increased at 12 h after treatment with 0.1 and 0.5 mM melatonin (Fig. 3e). *OsPR1a* expression was significantly upregulated at 6 h after treatment with 0.025 and 0.1 mM melatonin, with peak expression observed at 6 h after 0.1 mM treatment (Fig. 3g). Conversely, *OsJAZ8* expression was significantly decreased after treatment with 0.1 and 0.5 mM melatonin (Fig. 3h). These results indicate that melatonin activates rice immune responses, including phosphorylation of MAPKs, up-regulation of defense-related genes, and down-regulation of a repressor of JA signaling, thereby potentiating rice immunity against *M. oryzae*.

### Melatonin-Induced Maximal Expression of Defense-Related Genes Requires *OsCEBiP *and *OsCERK1*

**Fig. 4:**
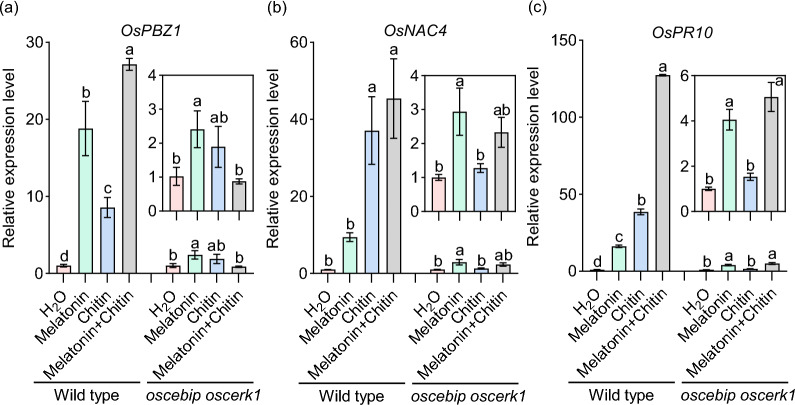
Melatonin-induced maximal induction of defense-related genes requires *OsCEBiP* and *OsCERK1.* **a**–**c** RT-qPCR was employed to analyze the expression of *OsPBZ1* (**a**), *OsNAC4* (**b**), and *OsPR10* (**c**) following treatment with H_2_O, melatonin, chitin, and a combination of chitin and melatonin in the *oscebip oscerk1* double mutant and wild-type control. The data are presented as means ± SE (n = 3). Lowercase letters in the figure indicate statistically significant differences (*P* < 0.05, one-way ANOVA with Tukey’s test). All experiments were performed three times with similar results

To investigate whether melatonin-enhanced immunity requires chitin receptors, we treated the *oscebip oscerk1* double mutant and wild-type control with H₂O, melatonin, chitin, and a combination of melatonin and chitin. In wild-type, the expression of *OsPBZ1*, *OsNAC4*, and *OsPR10* was significantly upregulated by melatonin or chitin compared to H₂O (Fig. 4). Notably, co-treatment with melatonin and chitin resulted in a synergistic increase in the expression of *OsPBZ1* and *OsPR10* compared to either treatment alone (Fig. 4). In contrast, chitin-induced upregulation of *OsPBZ1*, *OsNAC4*, and *OsPR10* was abolished in the *oscebip oscerk1* mutant (Fig. 4). Melatonin-induced upregulation of *OsPBZ1*, *OsNAC4*, and *OsPR10* was substantially reduced in the mutant compared to the wild-type control (Fig. 4). Interestingly, although melatonin-induced upregulation of defense-related gene expression was diminished in the mutant, the expression of *OsPBZ1*, *OsNAC4*, and *OsPR10* remained significantly higher than those in H₂O or chitin treatment. This may be attributed to immune responses mediated by alternative receptors capable of perceiving melatonin. These results indicated that melatonin-induced maximal induction of defense-related genes requires *OsCEBiP* and *OsCERK1*.

### Melatonin Inhibits *M. Oryzae* Growth

**Fig. 5:**
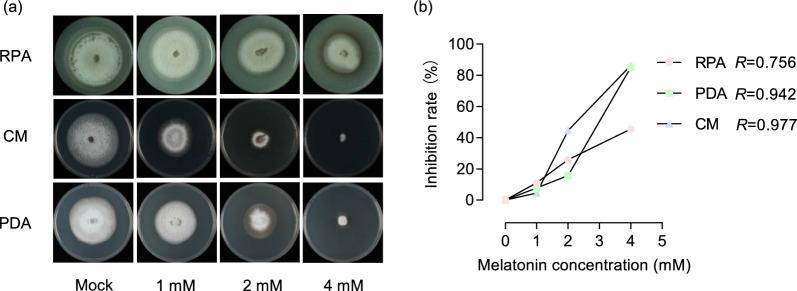
Melatonin inhibits the growth of *M. oryzae*. **a** Inhibitory effects of melatonin on the mycelial growth of *M. oryzae* strain GZ8 were assessed on RPA, CM, and PDA media containing melatonin at the indicated concentrations. Data were collected at 10 days post-inoculation (dpi). **b** The inhibition rate increased with rising melatonin concentrations, with the corresponding Pearson correlation coefficients denoted as *R.* In this figure, lowercase letters indicate statistically significant differences (*P* < 0.05, one-way ANOVA with Tukey’s test). All experiments were performed three times with similar results

To investigate whether melatonin directly mounts any influences on *M. oryzae*, we examined the growth of the *M*. *oryzae* GZ8 strain on three different mediums: complete medium (CM), rice powder agar (RPA) medium, and potato dextrose agar (PDA) medium, each supplemented with varying concentrations of melatonin. The mycelial growth of GZ8 was significantly inhibited by melatonin in all the three media in a concentration-dependent manner, as the colonies decreased along with the increase of the melatonin concentration (Fig. 5 and Fig. S2). Notably, the most substantial inhibitory effect on GZ8 growth was observed when melatonin was added in CM medium, followed by PDA medium, with the weakest inhibition detected in RPA medium (Fig. 5a). Consistently, the inhibition rate increased with rising melatonin concentration (Fig. 5b). These results indicate that melatonin mounts direct inhibition on the growth of *M. oryzae*.

### Melatonin Inhibits Spore Production and Germination of *M. Oryzae*

**Fig. 6:**
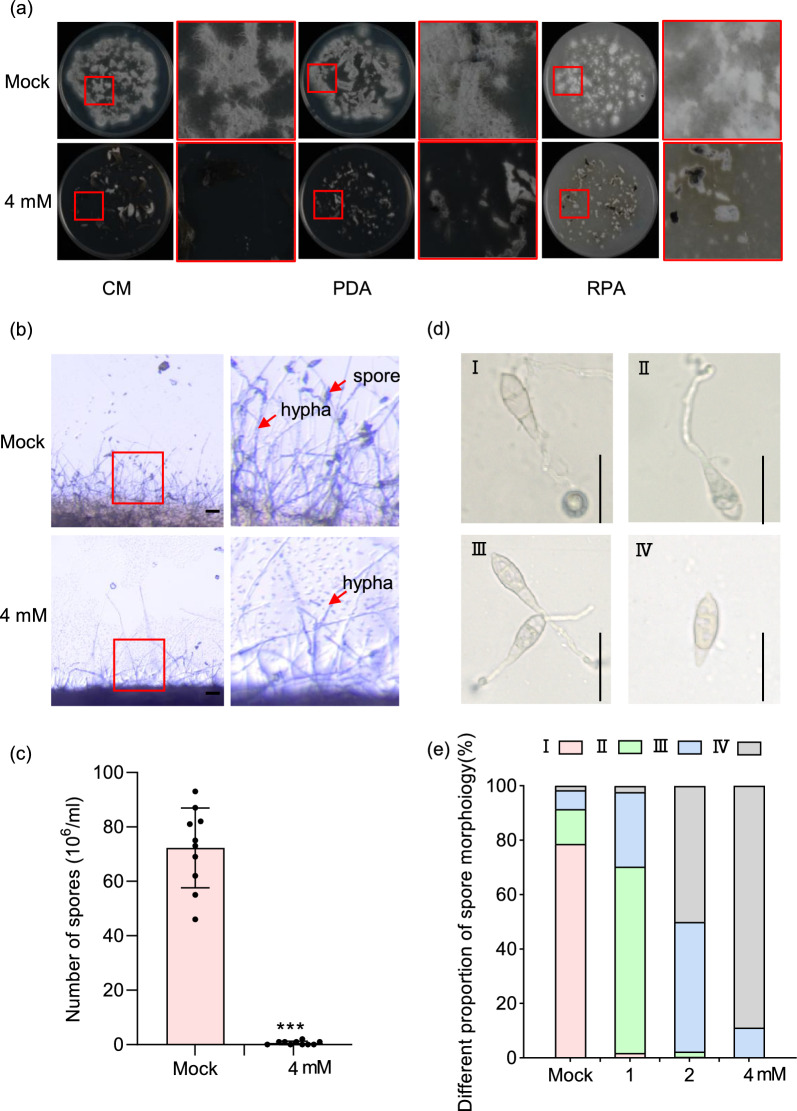
Melatonin inhibits spore production and germination of *M. oryzae*. **a** The spore production of the GZ8 strain was observed on RPA, CM, and PDA media supplied with/without melatonin. **b** A zoomed-in view of spore production on CM medium with/without melatonin. **c** Quantitative analysis of the blast spore number on CM medium containing either 0 mM or 4 mM melatonin as shown in **a**. Asterisks indicate the statistically significant difference (*P* < 0.001, Student’s *t*-test). **d** Morphological classification of spore germination observed under microscope after melatonin treatment. **e** Melatonin inhibits the germination of the blast spores. Shown is the percentage of spores in different germination states after treatment with different concentrations of melatonin

To further elucidate the effects of melatonin on *M. oryzae*, we examined the sporulation by evenly inoculating the GZ8 strain collected from CM medium onto RPA, CM, and PDA media that contain 0 or 4 mM melatonin. Following four days of continuous illumination, we observed that GZ8 sporulated normally on melatonin-free media, whereas sporulation was severely impaired on the media containing 4 mM melatonin (Fig. 6a). Microscopic examination demonstrated that a large number of spores were produced in the CM medium, whereas 4 mM melatonin completely abolished sporulation (Fig. 6b). Consistently, quantitative analysis indicated that melatonin significantly inhibited spore production (Fig. 6c).

To determine the effects of melatonin on spore germination, we treated GZ8 spores with four concentrations of melatonin, namely 0, 1, 2, and 4 mM, on hydrophobic coverslips at room temperature. Spore germination displayed four distinct types (Fig. 6d): (1) spores with prominent appressoria, (2) spores without appressoria but with long germ tubes, (3) spores with shorter germ tubes than type 2, and (4) spores with no signs of germination. In the absence of melatonin, type 1 accounted for 78.6% of the population, while type 4 accounted for only 1.6%. Notably, with the increasing melatonin concentrations, there was an obvious shift towards increased proportions of type 2, 3, and 4, indicating remarkable reductions in spore germination (Fig. 6e). Specifically, under 1 mM melatonin treatment, type 2 was the most prevalent (68.4%), followed by type 3 (27.4%). Under 2 mM melatonin treatment, type 3 and type 4 were proportionally equal (47.7% and 50%, respectively). Under 4 mM melatonin treatment, almost no spores germinated (Fig. 6e).

Taken together, we demonstrated that melatonin inhibits conidiation, spore germination, germ tube elongation, and appressorium formation, with higher melatonin concentrations exerting more pronounced inhibitory effects.

### Melatonin Induces Cell Death and Reduces ROS Accumulation in *M. Oryzae*

**Fig. 7:**
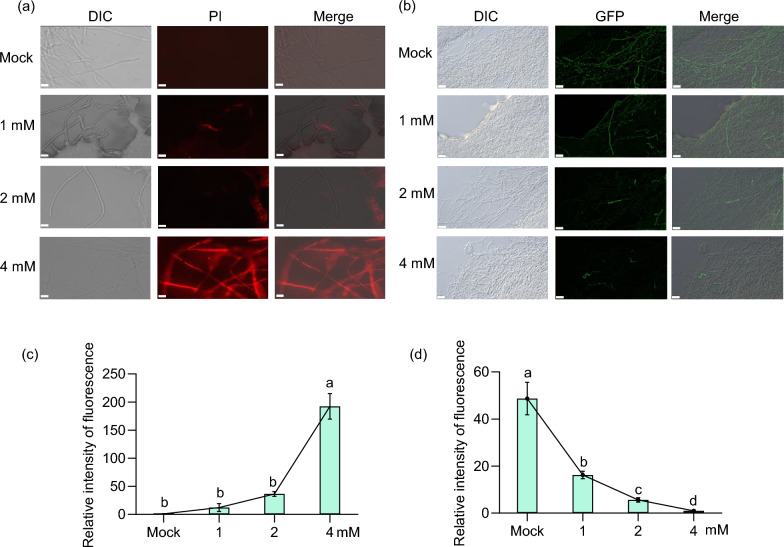
Melatonin induces mycelial death and inhibits reactive oxygen species (ROS) production in *M. oryzae*. **a**, **b** The mycelia of *M. oryzae* were treated with 0, 1, 2, and 4 mM melatonin for 8 h, followed by staining with PI and DCFH-DA dyes. After washing with distilled water, mycelial death (**a**) and ROS production (**b**) were observed under a fluorescence microscope. Scale bar: 5 μm (**a**) and 20 μm (**b**). **c**, **d** The optical density in **a** and **b** was quantitatively analyzed, respectively. The data are represented by means ± SE (n = 3). Lowercase letters indicate the statistically significant difference (*P* < 0.05, one-way ANOVA with Tukey’s test). All the experiments were conducted three times with similar results

A previous report demonstrated that melatonin induces cell death of a bacterial pathogen (Chen et al. 2019). To explore whether melatonin has similar roles in inducing cell death of *M. oryzae*, we treated Zhong10-8–14 mycelia with different concentrations of melatonin. As expected, increased melatonin concentrations enhanced mycelial death, with maximum mycelial death observed at 4 mM (Fig. 7a, c). Conversely, ROS production remarkably decreased in Zhong10-8–14 mycelia as melatonin concentrations increased, with virtually undetectable levels of ROS at 4 mM melatonin (Fig. 7b, d). This finding aligns with the established antioxidant properties of melatonin, which is reported to scavenge ROS (Liu et al. 2020). These results indicate that melatonin induces cell death and reduces the ROS accumulation in blast fungus.

## Discussion

Currently, conventional chemical fungicides, such as triazole and strobilurin, are widely employed to control rice blast (Kongcharoen et al. 2020). However, the extensive use of these fungicides raises concerns about their potential impact on human health and the environment (Rani et al. 2021). Biopesticides, derived from biological sources like microorganisms, plant extracts, or natural products, offer great potential due to their minimal environmental impact and reduced harm to non-target organisms (Seiber et al. 2014). Melatonin is a pleiotropic molecule and is widely found in animals, plants, and microorganisms (Jones et al. 2007; Arnao and Hernández-Ruiz 2015). Melatonin is commonly found in the roots, stems, leaves, flowers, fruits, and seeds of plants and is concentrated in the vigorously growing parts of these organs (Erland et al. 2018; Lee et al. 2019). Here, we found that melatonin biosynthesis-related genes were induced by *M. oryzae* infection (Fig. 1 and Fig. S1), and melatonin treatment reduces rice blast disease severity (Fig. 2). Consistent with the reduction of blast disease severity, melatonin activates the phosphorylation of MAPK3 and MAPK6 and up-regulates the expression of the defense-related genes (Fig. 3). Notably, melatonin-induced maximal induction of defense-related genes requires the chitin receptors *OsCEBiP* and *OsCERK1*, implying a crosstalk between chitin-triggered immunity and melatonin signaling (Fig. 4). In addition, the application of melatonin could significantly inhibit the hyphal growth of *M. oryzae* (Fig. 5 and Fig. S2) and also inhibit the sporulation and spore germination of *M. oryzae* (Fig. 6). Furthermore, melatonin treatment causes the death of *M. oryza*e hyphal cells and reduces the concentration of H_2_O_2_ (Fig. 7).

Melatonin reduces blast disease severity by rapidly activating immunity such as the phosphorylation of MAPK3 and MAPK6, and up-regulation of defense-related genes. Recent transcriptomic analysis of *Lilium* treated with melatonin revealed that differentially expressed genes were primarily involved in plant-pathogen interactions, hormone signal transduction, MAPK pathways, phenylpropanoid biosynthesis, and phenylalanine metabolism. Notably, genes associated with the MAPK pathway showed the most variation, indicating that melatonin enhances *Lilium* resistance to *Botrytis elliptica* by regulating the MAPK pathway (Xie et al. 2022). Similarly, in the rice cultivar LTH, melatonin application rapidly triggered strong phosphorylation of MAPK3/6 (Fig. 3a), indicating activation of the MAPK signaling pathway. In addition, previous studies have shown that melatonin enhances the plant’s defense response by regulating the expression of defense-related genes. Similarly, in cherry tomato, melatonin induces ROS bursts and upregulates defense-related genes, including *SlWRKY70*, *SlNPR1*, *SlTGA5*, *SlPR1*, *SlPR2*, and *SlGLU*, boosting immunity to *Botrytis cinerea* (Li et al. 2022). In this study, 0.5 mM melatonin significantly upregulated the expression of *OsCEBiP*, *OsPBZ1*, *OsPR10*, and *OsNAC4* (Fig. 3c–f), while 0.1 mM melatonin significantly upregulated *OsPR1a* (Fig. 3g). The peak expression levels of *OsNAC4* and *OsPR1a* occurred six hours post-treatment, suggesting a temporal specificity in melatonin-mediated gene regulation. Additionally, melatonin treatment downregulated *OsJAZ8*, with downregulation becoming more pronounced as melatonin concentrations increased (Fig. 3h). These findings collectively indicate that melatonin orchestrates the regulation of defense-related genes, with distinct temporal and intensity responses among various genes. Melatonin-induced activation of MAPK and defense-related genes in rice warrants further investigation to unravel the underlying mechanisms.

Melatonin effectively inhibits the growth of hyphae, spore production, and spore germination in *M. oryza*e (Figs. 5, 6), and it is widely found in nature, making it an excellent choice as a biopesticide. Biological pesticides inhibit the growth of pathogenic microorganisms, thereby suppressing their proliferation within the host. For example, ethylicin treatment induces secretion of extracellular polysaccharides and enzymes of *Xanthomonas oryzae* pv. *oryzicola*, disrupting its cell membrane and oxidative phosphorylation pathway, thereby inhibiting its growth (Huang et al. 2023). Physcion suppresses *M. oryzae* infection by inhibiting mycelial growth, conidial germination, and appressorium formation (Wang et al. 2016). 5-methoxyindole, a melatonin homolog at 4 mM concentration, effectively inhibits *Fusarium graminearum* by disrupting mycelial growth and spore germination (Kong et al. 2021). This study demonstrated that melatonin effectively inhibits *M. oryzae* proliferation (Figs. 5, 6, 7). Our results showed that melatonin treatment significantly reduced H_2_O_2_ accumulation in *M. oryzae* mycelia (Fig. 7b, d). While melatonin is known as a ROS scavenger (Arnao and Hernández-Ruiz 2015), exogenous melatonin has also been reported to induce ROS production (Li et al. 2022). The mechanism of hyphal death caused by melatonin is not clear at present, and whether melatonin reduces the accumulation of ROS of *M. oryzae* affects its pathogenic ability is not clear, which needs to be explored in subsequent experiments. Recent research has demonstrated that melatonin can efficiently penetrate fungal cells and colocalize with the mitogen-activated protein kinase Mps1. Melatonin binds directly to Mps1 and inhibits its phosphorylation, thereby disrupting septin-ring formation and impairing appressorium development (Li et al. 2023; Eisermann and Talbot 2024). Melatonin is an environmentally safe natural product, modifying melatonin provides a strategy for developing antifungal agents (Cai et al. 2024). Moreover, melatonin can be combined with other fungicides to enhance their efficacy. For example, the combination of melatonin and Infinito showed greater inhibition of *Phytophthora infestans* than either treatment alone (Zhang et al. 2017). Melatonin and isoprothiolane synergistically interfere with lipid metabolism by interacting with and regulating the predicted isocitrate lyase-encoding gene *MoIcl1* and can be used to reduce the dosage and residual level of isoprothiolane to control *M. oryzae* (Bi et al. 2023). Therefore, melatonin emerges as a promising adjunctive antifungal agent, as it not only directly impairs fungal growth but also augments the efficacy of existing antifungal treatments when employed in combination.

The minimum concentrations of melatonin required to induce plant defense are distinct from those needed to inhibit pathogen growth. Specifically, low concentrations of melatonin effectively trigger plant defense responses. For example, treating leaves with 1 μM melatonin in *Arabidopsis thaliana* rapidly induces the expression of defense genes such as *PR1*, *PDF1.2*, *ICS1*, *ACS6*, *GST1*, *APX1*, and *VSP1*, enhancing plant immunity against *Pseudomonas syringae* (Lee et al. 2014). Consistently, as low as 0.1 μM melatonin is sufficient to activate the MAPK signaling cascade in Arabidopsis and tobacco (Lee and Back 2016). Analogously, in this study, 25 μM melatonin activated the MAPK cascade and upregulated the expression of defense-related genes. In contrast, higher concentrations of melatonin are necessary to inhibit pathogen growth directly. For example, 4 mM melatonin is most effective at inhibiting the formation and germination of *F. graminearum* conidia (Kong et al. 2021). Moreover, the mycelial growth of *Phytophthora capsici* was significantly inhibited by 5–8 mM melatonin (Mandal et al. 2018). Similarly, this study showed that concentrations ranging from 1 to 4 mM of melatonin significantly inhibited *M. oryzae* growth and conidial germination, and induced mycelial death. Therefore, the effective concentrations of melatonin for activating plant defense responses versus inhibiting pathogen growth differ significantly.

## Conclusion

**Fig. 8:**
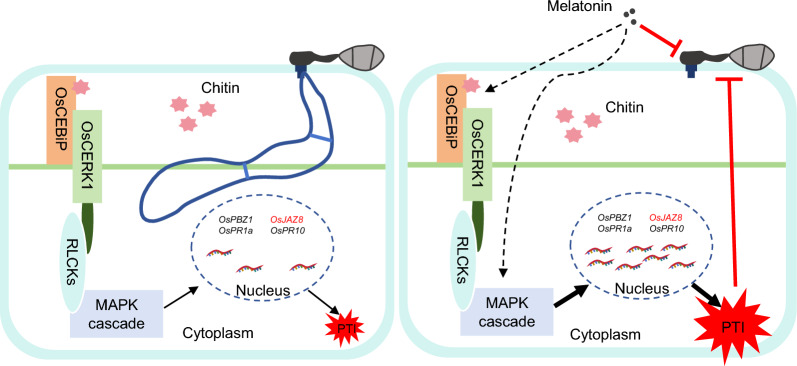
Melatonin enhances rice immunity and inhibits the growth of *M. oryzae*. Proposed model illustrating the role of melatonin in rice and blast interaction. In the absence of melatonin, the chitin from the blast fungus triggers a relatively weak pattern-triggered immunity (PTI) in rice. However, the presence of melatonin induces a robust immune response in rice while also inhibiting the growth of the blast fungus

Overall, melatonin elicits a robust immune response in rice while concurrently inhibiting the growth and development of the rice blast fungus. Thus, melatonin performs dual functions in both the plant and the pathogen, enhancing rice resistance to blast disease by augmenting plant immunity and suppressing pathogen development (Fig. 8). This study adds robust evidence supporting the development of melatonin-based eco-friendly biopesticides.

## Supplementary Information

Additional file 1: Table S1. The primers used in this study

Additional file 2: Figure S1. The expression of melatonin biosynthesis-related genes was upregulated in response to *M. oryzae* inoculation. a-f, RT-qPCR was employed to analyze the expression patterns of *OsTDC4*, *OsTDC5*, *OsT5H1*, *OsSNAT1*, *OsASMT13*, and *OsASMT15*in LTH following spray inoculation with *M. oryzae* at 0, 24, 48, 72, 96, 120, 144, and 168 h. The data are presented as means ± SE. Lowercase letters in the figure indicate statistically significant differences. All experiments were performed three times with similar results. Figure S2. Inhibitory effects of melatonin on mycelial growth of *M. oryzae*. The colony radius and inhibition rate of the GZ8 strain were quantitatively analyzed in Fig. 3a. The data are represented by means ± SE. Lowercase letters indicate the statistically significant difference. All the experiments were conducted three times with similar results.

## Data Availability

No datasets were generated or analysed during the current study.

## Abbreviations

MAPK: Mitogen-activated protein kinase
*M. oryzae*: *Magnaporthe oryzae*
AgNPs: Silver nanoparticles
SAN: Sanguinarine
ROS: Reactive oxygen species
*CBFs*: C-repeat binding factors
*DREBs*: *Drought Response Element Binding factors*
*COR15a*: *Cold-regulated 15a*
*CAMTA1*: *Calmodulin-binding transcription activator 1*
*ZAT10*: *C*_*2*_ *H*_*2*_ *-type zinc finger transcription factor 10*
*ZAT12*: *C*_*2*_ *H*_*2*_ *-type zinc finger transcription factor 12*
SA: Salicylic acid
JA: Jasmonic acid
ET: Ethylene
LTH: Lijiangxin Tuan Heigu
GZ8: EGFP-tagged *M. oryzae* strain Zhong10-8-14
CM: Complete medium
RT-qPCR: Real-time quantitative polymerase chain reaction
*UBI*: *ubiquitin*
RPA: Rice powder agar
PDA: Potato dextrose agar
YEPD: Yeast extract peptone dextrose
PI: Propidium iodide
DCFH-DA: 2,7-Dichlorofluorescein diacetate
