# Supplementary figures and images for: Melatonin Enhances Blast Disease Resistance via Inducing Rice Immunity and Inhibits the Growth of the Magnaporthe Oryzae
Source: Rice (N Y). 2025 Jul 19;18:69. doi: 10.1186/s12284-025-00824-1 (PMC12276192; doi:10.1186/s12284-025-00824-1)

## Slide 1
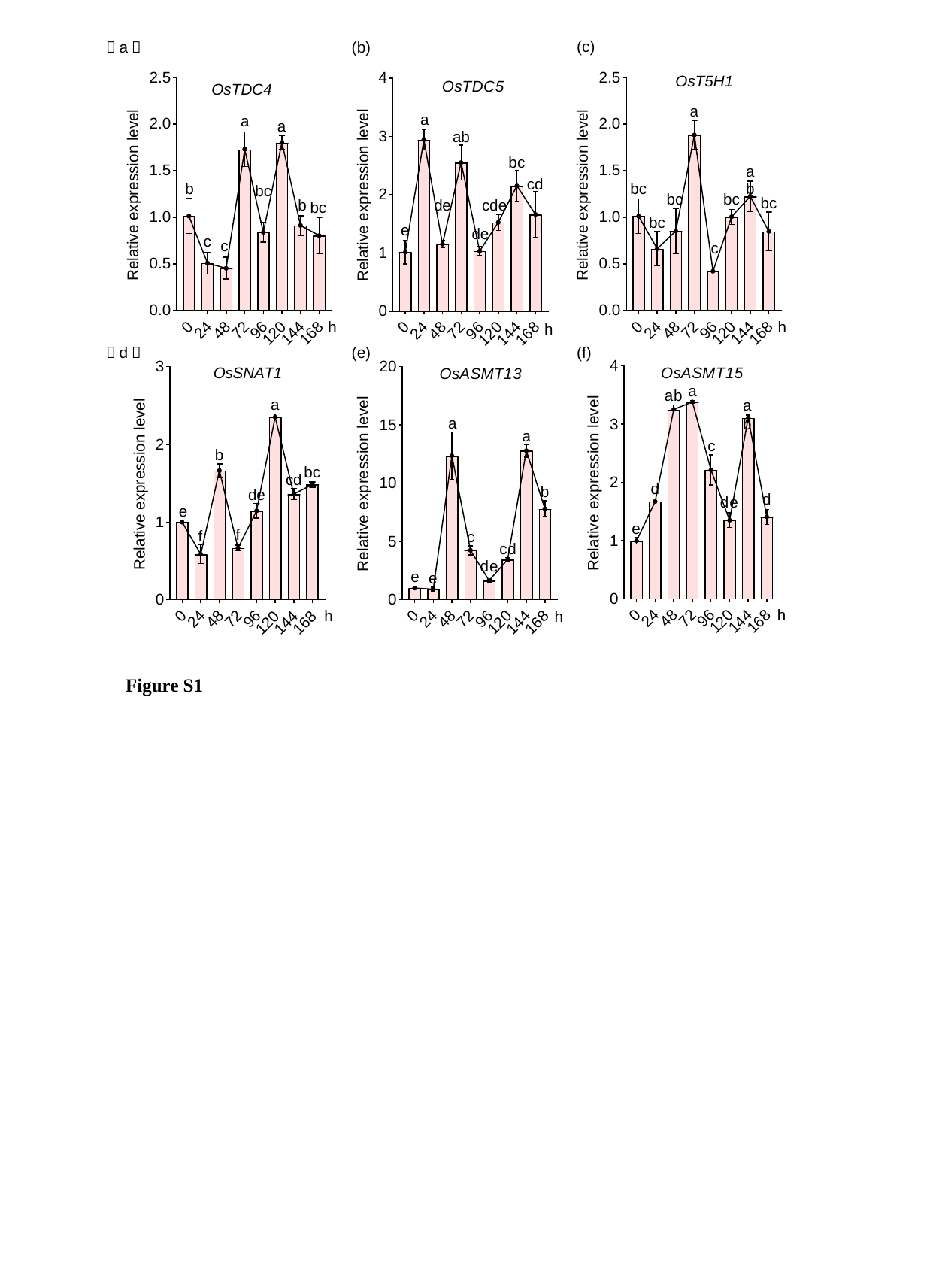

(c)
（a）
(b)
(f)
（d）
(e)
Figure S1

## Slide 2
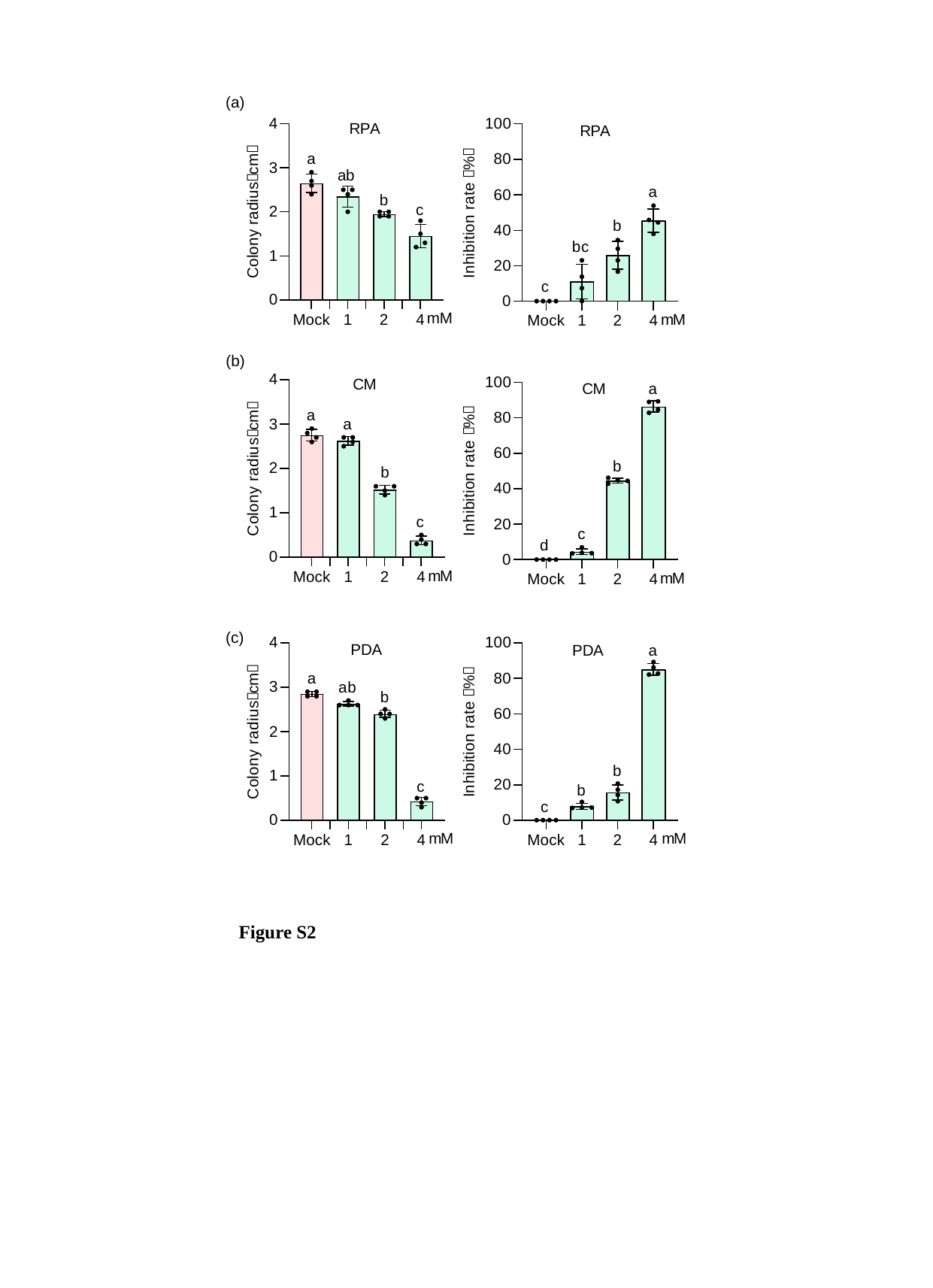

(a)
(b)
(c)
Figure S2

Supplement: Supplementary file 2 — Additional file 2: Figure S1. The expression of melatonin biosynthesis-related genes was upregulated in response to M. oryzae inoculation. a-f, RT-qPCR was employed to analyze the expression patterns of OsTDC4, OsTDC5, OsT5H1, OsSNAT1, OsASMT13, and OsASMT15in LTH following spray inoculation with M. oryzae at 0, 24, 48, 72, 96, 120, 144, and 168 h. The data are presented as means ± SE. Lowercase letters in the figure indicate statistically significant differences. All experiments were performed three times with similar results. Figure S2. Inhibitory effects of melatonin on mycelial growth of M. oryzae. The colony radius and inhibition rate of the GZ8 strain were quantitatively analyzed in Fig. 3a. The data are represented by means ± SE. Lowercase letters indicate the statistically significant difference. All the experiments were conducted three times with similar results. [file 12284_2025_824_MOESM2_ESM.pptx]
